# The NIH-NIAID Schistosomiasis Resource Center

**DOI:** 10.1371/journal.pntd.0000267

**Published:** 2008-07-30

**Authors:** Fred A. Lewis, Yung-san Liang, Nithya Raghavan, Matty Knight

**Affiliations:** Biomedical Research Institute, Rockville, Maryland, United States of America; Queensland Institute of Medical Research, Australia

## Abstract

A bench scientist studying schistosomiasis must make a large commitment to maintain the parasite's life cycle, which necessarily involves a mammalian (definitive) host and the appropriate species of snail (intermediate host). This is often a difficult and expensive commitment to make, especially in the face of ever-tightening funds for tropical disease research. In addition to funding concerns, investigators usually face additional problems in the allocation of sufficient lab space to this effort (especially for snail rearing) and the limited availability of personnel experienced with life cycle upkeep. These problems can be especially daunting for the new investigator entering the field. Over 40 years ago, the National Institutes of Health–National Institute of Allergy and Infectious Diseases (NIH-NIAID) had the foresight to establish a resource from which investigators could obtain various schistosome life stages without having to expend the effort and funds necessary to maintain the entire life cycle on their own. This centralized resource translated into cost savings to both NIH-NIAID and to principal investigators by freeing up personnel costs on grants and allowing investigators to divert more funds to targeted research goals. Many investigators, especially those new to the field of tropical medicine, are only vaguely, if at all, aware of the scope of materials and support provided by this resource. This review is intended to help remedy that situation. Following a short history of the contract, we will give a brief description of the schistosome species provided, provide an estimate of the impact the resource has had on the research community, and describe some new additions and potential benefits the resource center might have for the ever-changing research interests of investigators.

## History

**Figure 1:**
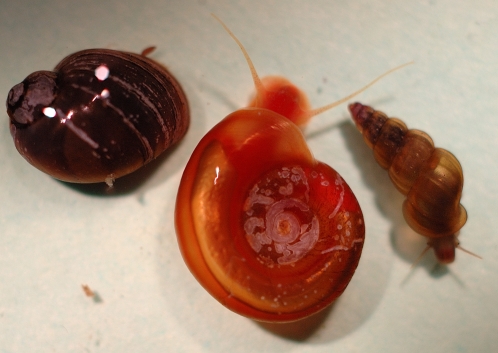
The Three Species of Intermediate Hosts That Are Provided from The Schistosomiasis Center. From left, *Bulinus truncatus truncatus* (host for *S. haematobium*), *Biomphalaria glabrata* (host for *S. mansoni*), and *Oncomelania hupensis hupensis* (intermediate host for the Chinese isolate of *S. japonicum*; other subspecies of *O. hupensis* are also available). For size comparisons, the *B. glabrata* shown is approximately 5 mm in shell diameter.

Our core resource laboratory maintains the three schistosome species that inflict the greatest human disease burden: *Schistosoma mansoni*, S. *haematobium*, and *S. japonicum*. Figure 1 shows the three species of snails (intermediate hosts) used for maintaining the three schistosome species above.

National Institutes of Health–National Institute of Allergy and Infectious Diseases (NIH-NIAID) funding to support this effort began in 1967, with the first contract award going to the University of Michigan. These early years were especially important for developing optimal conditions for lab maintenance of the very fastidious *Oncomelania* sp. hosts for *S. japonicum* [1]–[4]. In subsequent years, the resource center was maintained at the University of Massachusetts–Lowell (1977–1995), and, since 1995, at the Biomedical Research Institute (BRI; Rockville, Maryland, United States of America). The original intent of the resource—to provide living schistosome life cycle stages to research investigators at no cost— still comprises the dominant portion of the effort. As will be discussed in more detail later, the work has been expanded in the past few years to keep up with the increasingly diversified interests of researchers.

Aside from shipping charges, living life cycle stages for the schistosome species and strains are provided free of charge as prepatent infections in the intermediate snail hosts, or as early infections in the definitive hosts (mouse or hamster). Due to space and funding considerations, the contract does not allow BRI to house the snails or mammals after infection, throughout the entire prepatent snail stage, or throughout the immature-to-mature stage in the definitive hosts. Therefore, the recipient labs must make some commitment to house the snails and/or mammals beginning about 1 week after exposure to the parasite.

**Figure 2:**
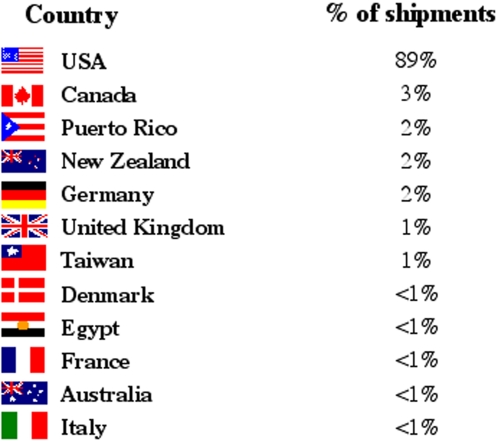
The Geographic Location of Recipient Laboratories of Snails and/or Mammals Provided by the NIH-NIAID Schistosomiasis Resource Center.

In any given year, the resource provides life cycle stages and other materials to between 35 and 50 separate laboratories worldwide, with the majority in the US (Figure 2). No distinction is made in Figure 2 regarding the proportion of snail versus mammal (mouse and/or hamster) shipments, but about two-thirds of total shipments are snails only. Approximately 39% of the recipient laboratories are located in graduate departments of universities, and 44% are in medical or veterinary schools. The remaining 17% are located in undergraduate institutions, where the investigators have active schistosomiasis research programs and/or use schistosomes as a teaching tool for biology and parasitology classes, taking advantage of the fact that schistosomes are good representative teaching models for helminth infections in general.

## Schistosome Species Provided

### *Schistosoma mansoni*

Of the three schistosome species provided by this resource, *S. mansoni* has been, by far, the subject of most schistosomiasis research. It is the easiest of the three major schistosome species to maintain in the laboratory and, not surprisingly, it is first in terms of sheer numbers of infected snails and mammals requested. It is also the only species of the three provided that is endemic in several countries of both the Eastern and Western hemispheres. Of great historical importance in expanding schistosomiasis research was the work in Puerto Rico and Brazil with *S. mansoni* in the 1950s and 1960s, and Puerto Rican isolates of *S. mansoni* still are used widely as representative strains in the *S. mansoni* research community. Our two Puerto Rican isolates of *S. mansoni* (NMRI and PR-1) are maintained in the planorbid snail *Biomphalaria glabrata*, which is the major intermediate host for *S. mansoni* in the Western hemisphere. The most commonly used *B. glabrata* snail stock for maintenance of *S. mansoni* arose from an albino mutant stock from research by Newton [5]. Not only did it prove to be highly susceptible to *S. mansoni*, but it also allowed investigators using a dissecting microscope to examine intramolluscan development of the parasite, otherwise made difficult by the normal black pigmentation typically found in snails isolated from the field.

### *Schistosoma japonicum*

Laboratory maintenance of *S. japonicum* is the most difficult of the three schistosome species provided by this resource. Workers experienced with this parasite's intermediate snail host (*Oncomelania* sp.) have long been frustrated by the difficulty of coercing this species to grow vigorously in laboratory settings [1]. Its breeding characteristics, size, and cercarial shedding behavior in the laboratory are vastly different from that of its *Biomphalaria* and *Bulinu*s counterparts. *S. japonicum*–induced pathology in the mouse, on a worm pair comparison, is considerably more pronounced than that of *S. mansoni* infections, with only one or two *S. japonicum* worm pairs producing dramatic granulomatous disease in the laboratory mouse by 7 weeks post-cercarial exposure. Two subspecies of *S. japonicum* are provided; one a Philippine isolate, the other from China. These two subspecies are provided either in *Oncomelania hupensis quadrasi* (for the Philippine isolate) or in *O. h. hupensis* (for the Chinese isolate).

### *Schistosoma haematobium*

Although *S. haematobium* is of wide-ranging clinical importance throughout much of Africa and the Middle East, significantly fewer requests have been made for this species from this resource than for *S. mansoni* or *S. japonicum*. This is probably because studying *S. haematobium* infections in the laboratory is complicated by the absence of a small laboratory animal model in which pathology resembles the human infection with this parasite. Through the years the standard mammalian laboratory host for *S. haematobium* has been the Syrian golden hamster. Although the parasite develops to maturity, the urogenital system of the hamster is not significantly involved in the disease. On the contrary, upon dissection, the involvement of the liver and intestinal tract resemble an infection with *S. mansoni*, and the majority of the adult worms can be found in the mesenteric venules, as with an *S. mansoni* infection. It is unfortunate for experimental studies that a more suitable small laboratory host has not been found that better approximates the infection in humans, since one of the more intriguing aspects of its infection in humans is its association with bladder cancer [6],[7]. It is our belief that, if for no other reason, this alone should give investigators even more incentive to study this important parasite. The snail hosts for laboratory maintenance of this parasite, members of the genus *Bulinus*, are relatively easy to propagate in the laboratory, and their requirements for successful maintenance are much like those for *Biomphalaria*. This resource provides an Egyptian isolate of *S. haematobium*, maintained in *Bulinus truncatus truncatus* snails and in hamsters.

## Impact of the Resource on the Schistosomiasis Research Community

It would be impossible to give an accurate number of publications in experimental schistosomiasis that have been made possible through the use of this resource over the 40-plus years of its existence. Certainly it would be in the thousands. At least two barometers, however, can give some idea of its impact on the schistosomiasis research community. Browsing through the past 10 years of presentations in experimental schistosomiasis from the annual meetings of The American Society of Tropical Medicine and Hygiene (ASTMH) shows that roughly 80% of those presentations were from laboratories that have relied upon material support from this resource. Another indication of its importance comes from PubMed searches. Over the last 3 years, approximately 90% of the experimental schistosomiasis papers are from laboratories relying on these materials.

## Other Services

In recent years, additions have been made to the contract to meet other needs in schistosomiasis research.

### Training

In the spring and fall of each year, our laboratory offers a training course in *S. mansoni* maintenance, allowing investigators to gain more experience with the various schistosome life stages and several of the procedures most common to schistosomiasis research laboratories. Our overriding concern is to help investigators avoid parasite-related problems that might lead to erroneous experimental outcomes—a common problem in many labs that handle these complex life cycles. An added benefit to the community is to establish standard, basic techniques to make each investigator's life cycles more productive. It is hoped that this effort will help investigators avoid, as much as possible, unproductive experiments due to some fundamental problem with the life cycle. Currently, each class is 2 days long and is limited to eight attendees per class. The attendees have ranged from experienced researchers and/or their students and technicians who maintain their own life cycles, to those who are new to the field of schistosomiasis research and lack prior hands-on experience with *S. mansoni*.

### Cryopreservation of Schistosome Strains

Techniques have been developed to cryopreserve schistosomules as a means of long-term storage, foregoing the need for continually passing the parasite through snail and mammalian hosts [8],[9]. Apart from the convenience of cryopreserving the parasites and restoring the strain only when needed, this technique can be of great practical benefit for studies such as those examining genetic changes in schistosomes as a result of several cycles of selection pressure under experimental conditions [10]. In addition, cryopreservation offers the possibility of storing large numbers of different field isolates for later comparative studies. Most of what is known about cryopreserving schistosomes comes from the work with *S. mansoni*, and with present techniques, approximately 2%–5% of the cryopreserved and thawed schistosomules can mature in mice. Although this is considerably lower than the typical 40%–50% maturation rate of skin-penetrating cercariae in mice, it has been our experience that if at least 20,000 cercariae are in the starting (pre-cryopreserved) population, recovery is usually sufficient to re-establish the strain once thawed [11]. BRI offers the service of cryopreserving individual *S. mansoni* strains, keeping them in liquid nitrogen for long-term storage, and re-establishing the strain in mice when requested.

### The Schistosome Related Reagent Repository (SR3)

The Schistosome Related Reagent Repository (SR3) was launched in 2003 to serve as a central facility for the collection and long-term storage of schistosome and snail–host related reagents. This allows scientists: 1) improved access to parasite, snail, and related reagents; and 2) access to standardized reagents in a renewable form. This repository was developed to provide community-wide access to standardized and well-characterized materials that are generated and deposited by other investigators. At this time, the SR3 maintains a relatively small collection of *S. mansoni* cDNA libraries, and several *B. glabrata*–related molecular reagents (primarily cDNA libraries from various tissues). We anticipate that the repository will soon include a wider range of schistosome- and snail-related reagents such as: (a) genomic libraries, cDNA libraries, glycerol stocks and bacterial stabs of recombinant clones, recombinant plasmid DNA, oligonucleotide probes, and PCR primers; (b) antisera against schistosome products (storage only); and (c) reagents added on an as-need basis, such as high-density filters of gridded bacterial artificial chromosome, expressed sequence tag, and cosmid libraries.

The SR3 also continuously maintains, in culture, the *B. glabrata* embryonic (*Bge*) cell line, derived from the susceptible M-line snail [12]. This cell line is of great benefit for exploring molecular signaling events underlying the host–parasite interaction of *S. mansoni*. The cell line also has application for karyotyping and mapping the snail genome as part of the genome sequencing project. Hands-on training is available to help researchers with culturing this cell line and setting up co-cultures with sporocysts.

## Conclusion

From its beginning, the NIH-NIAID-supported core schistosomiasis resource center has been a major driving force in the progress of schistosomiasis research. Not only has this been important in helping to decrease the health burden of schistosomiasis, but investigators are increasingly appreciating that a schistosome infection provides a good model system for studying a variety of inflammatory, allergic, and granulomatous diseases. For example, *S. mansoni* infection in mice provides an elegant model for helping unravel contributions of separate helper T cell populations (Th1 and Th2) in the development of asthma, allergic inflammation, and fibrosis [13]. Thus, laboratory studies of schistosomiasis are leading to advances in medicine that likely will have implications far beyond that of controlling the disease itself. Availability of this schistosome resource thus can serve as a foundation for individuals, not only in tropical medicine research, but in many diverse areas of basic medical research.

**Box 1. Summary Points**

1. For over 40 years, NIH-NIAID has supported a schistosome resource center to provide research material, free of charge, to principal investigators.
2. To improve efficiency, conditions have been developed to simultaneously house *S. mansoni*, *S. haematobium*, and *S. japonicum*.
3. The resource facility has been instrumental in creating a uniformity of source and standardization of study protocols.
4. The resource has been expanded in recent years to house schistosome-related molecular resources for genomic and proteomic research.
5. For capacity strengthening, training elements exist for established investigators and for those new to the field.

Web sites that can be accessed for information on provisions of the schistosome life cycle stages, schistosome-related molecular reagents, and other repository activities funded by NIH-NIAID are:

- NIAID Schistosomiasis Resource Center, http://www.schisto-resource.org/
- Schistosome Related Reagent Repository (SR3), http://www.afbr-bri.com/sr3/
- NIAID Research and Development Contracts, http://www.niaid.nih.gov/contract/

**Box 2. Testimonials from Scientists Receiving Materials from the Schistosomiasis Resource Center**

*As a relative newcomer to the field, this resource has been essential in helping us establish our research program. The training element and continued support have been invaluable for our technicians and graduate students.* (Stephen Davies, USUHS, Bethesda, Maryland, United States of America)
*Without this resource our research would have been impossible. The patient training, troubleshooting, and help during emergency needs are appreciated from the entire community of schistosomiasis researchers.* (Miguel Stadecker, Tufts University, Boston, Massachusetts, United States of America)
*This facility has been of immense help to our research on Schistosoma japonicum, and has provided extensive training in life cycle maintenance with this parasite. Serving such a large group of schistosome researchers, it is truly an invaluable resource and something that must be continued.* (Don McManus, Queensland Institute of Medical Research, Brisbane, Queensland, Australia)
*This is a highly valued resource to the entire blood fluke community. The consistency of mouse infections has always been highly uniform, such that we never have to be concerned with “batch-to-batch” variation in parasite yields or quality. The provision of supplemental numbers of snails during times of need has also been of great benefit and crucial to the success of our research program.* (Tim Yoshino, University of Wisconsin-Madison, Wisconsin, United States of America)
*My laboratory would not be able to perform studies on development of vaccines or immunoregulation without the life cycle support provided on this contract. Like clockwork, the snails are provided each month. A truly great resource.* (Don Harn, Harvard University, Boston, Massachusetts, United States of America)

**Box 3. Five Key Original Studies and Reviews Made Possible by the Materials/Stages from the NIAID Schistosomiasis Resource Center**

1. Boros DL, Warren KS (1970) Delayed hypersensitivity granuloma formation and dermal reaction induced and elicited by a soluble factor isolated from *Schistosoma mansoni* eggs. J Exp Med 132: 488–507.
*This study led to what we now know as the immune response to schistosome egg antigens being critical in driving the pathology of the disease.*
2. Georgi JR (1982) *Schistosoma mansoni*: quantification of skin penetration and early migration by differential external radioassay and autoradiography. Parasitol 84: 263–281.
*Application of this parasite tracking technique cleared up many of the issues of natural and vaccine-induced immunity in experimental animals.*
3. Pearce EJ, MacDonald AS (2002) The immunobiology of schistosomiasis. Nature Rev Immunol 2: 499–511.
*Comprehensive review of the immune response in schistosomiasis infections in the mammalian host. The complexities of the immune balance between the acute versus chronic phase responses are well described, and the role of Th1- and Th2-type responses in the search for vaccines is discussed.*
4. Loker ES, Bayne CJ (2001) Molecular studies of the molluscan response to digenean infection. In: Beck G, Sugumaran M, Cooper EL, editors. Phylogenetic perspectives on the vertebrate immune system. New York: Plenum Publishers. pp. 209–222.
*This review serves as groundwork for the burgeoning studies on the molecular phases of the intramolluscan development of digeneans, and in particular schistosomes.*
5. McCutchan TF, Simpson AJG, Mullins JA, Sher A, Nash TE, Lewis F, Richards C (1984) Differentiation of schistosomes by species, strain, and sex by using cloned DNA markers. Proc Nat Acad Sci USA 81: 889–893.
*Showed the feasibility of using molecular markers, in this case cloned ribosomal gene segments, for differentiation in population genetic studies for schistosomes.*
